# Introducing IsoMad, a compilation of isotopic datasets for Madagascar

**DOI:** 10.1038/s41597-024-03705-2

**Published:** 2024-08-09

**Authors:** Sean W. Hixon, Ricardo Fernandes, Antonin Andriamahaihavana, Andrea L. Baden, Marina B. Blanco, Guillaume Caulier, Melanie Dammhahn, Igor Eeckhaut, Timothy M. Eppley, Bruno Frédérich, Jörg U. Ganzhorn, Andrius Garbaras, Dean Gibson, Steven M. Goodman, Mitchell Irwin, Elizabeth A. Kelley, Loïc N. Michel, Gilles Lepoint, James E. Loudon, Laurent Mittelheiser, Jacques Rakotondranary, Delaïd C. Rasamisoa, Richard Rasolofonirina, Yedidya Ratovonamana, Josia Razafindramanana, Christoph Reisdorff, Matt Sponheimer, Lucas Terrana, Natalie Vasey, Brooke E. Crowley

**Affiliations:** 1 Oregon State University, Department of Integrative Biology, 4575 SW Research Way, Corvallis, OR 97333 USA; 2 Max Planck Institute for Geoanthropology, Department of Archaeology, Kahlaische Strasse 10, 07745 Jena, Germany; 3 University of Warsaw, Faculty of Archaeology, Department of Bioarchaeology, ul. Krakowskie Przedmieście 26/28, 00-927 Warszawa, Poland; 4 Masaryk University, Arne Faculty of Arts, Nováka 1, 602 00 Brno-střed, Czech Republic; 5 Princeton University, Climate Change and History Research Initiative, Princeton, NJ 08544 USA; 6 University of Antananarivo, Mention Zoologie et Biodiversité Animale, Antananarivo, 101 Madagascar; 7 San Diego Zoo Wildlife Alliance, Conservation Science & Wildlife Health, 15600 San Pasqual Valley Rd., Escondido, CA 92027 USA; 8 City University of New York (CUNY), Hunter College, Department of Anthropology, 695 Park Avenue, New York, NY 10065 USA; 9 City University of New York, The Graduate Center, Department of Anthropology, 595 Park Avenue, New York, NY 100065 USA; 10 The New York Consortium in Evolutionary Primatology (NYCEP), New York, USA; 11 Duke University, Department of Biology, Biological Sciences Building, 130 Science Drive, Durham, NC 27708 USA; 12 University of Mons, Biology of Marine Organisms and Biomimetics, 23 Place du Parc, 7000 Mons, Belgium; 13 Belaza Marine Station (IH.SM-UMONS-ULIEGE-ULB), Toliara, Madagascar; 14 University of Münster, Institute for Neurobiology and Behavioural Biology, Badestrasse 9, 48149 Münster, Germany; 15 Wildlife Madagascar, 2907 Shelter Island Drive, Suite 105, PMB 1024, San Diego, CA 92106 USA; 16 Portland State University, Department of Anthropology, 141 Cramer Hall, 1721 SW Broadway, Portland, OR 97201 USA; 17 University of Liège, Laboratory of Evolutionary Ecology, 11 Allée du six ao û t, Building B6c, University of Liège, 4000 Liège, Belgium; 18 University of Hamburg, Department of Biology, Martin-Luther-King Platz 3, 20146 Hamburg, Germany; 19 Center for Physical Sciences and Technology, Isotope Research Laboratory, Savanoriu av. 231, Vilnius, Lithuania; 20 Field Museum of Natural History, Negaunee Integrative Research Center, 1400 South DuSable Shore Drive, Chicago, IL 60605 USA; 21 Association Vahatra, BP 3972, Antananarivo, 101 Madagascar; 22 Northern Illinois University, Department of Anthropology, 1425 W Lincoln Hwy, DeKalb, IL 60115 USA; 23 NGO Sadabe, Lot AB64bis, Ankadindravola, Antananarivo, 105 Madagascar; 24 Saint Louis Zoo, 1 Government Drive, St. Louis, MO 63110 USA; 25 University of Liège, Department of Animal Systematics and Diversity, 11 Allée du six ao û t, Building B6c, University of Liège, 4000 Liège, Belgium; 26 University of Liège, Laboratory of Trophic and Isotope Ecology, 11 Allée du six ao û t, Building B6c, University of Liège, 4000 Liège, Belgium; 27 East Carolina University, Department of Anthropology, E 5th Street, Greenville, NC 27858 USA; 28 University of Antananarivo, Mention Anthropobiologie et Développement Durable, BP 906, Antananarivo, 101 Madagascar; 29 Wildlife Madagascar, Antananarivo, 101 Madagascar; 30 University of Toliara, Institut d’Halieutique et de Sciences Marine, 48B042, Rue Dr. Rabesandratana HD, BP 141, Toliara, 601 Madagascar; 31 University of Antananarivo, Department of Biology and Plant Ecology, BP 906, Antananarivo, 101 Madagascar; 32 IMPACT Madagascar, Antananarivo, 101 Madagascar; 33 University of Colorado, Boulder, Department of Anthropology, UCB 244, Boulder, CO 80309 USA; 34 Natural History Museum and Vivarium of Tournai, Cour d’Honneur de l’Hôtel de Ville 52, 7500 Tournai, Belgium; 35 University of Cincinnati, Department of Geosciences, 500 Geology Physics Building, 345 Clifton Court, Cincinnati, OH 45221-0013 USA; 36 University of Cincinnati, Department of Anthropology, 481 Braunstein Hall, 345 Clifton Court, Cincinnati, OH 45221-0380 USA

**Keywords:** Stable isotope analysis, Biogeochemistry, Stable isotope analysis, Plant ecology, Tropical ecology

## Abstract

We present the first open-access, island-wide isotopic database (IsoMad) for modern biologically relevant materials collected on Madagascar within the past 150 years from both terrestrial and nearshore marine environments. Isotopic research on the island has increasingly helped with biological studies of endemic organisms, including evaluating foraging niches and investigating factors that affect the spatial distribution and abundance of species. The IsoMad database should facilitate future work by making it easy for researchers to access existing data (even for those who are relatively unfamiliar with the literature) and identify both research gaps and opportunities for using various isotope systems to answer research questions. We also hope that this database will encourage full data reporting in future publications.

## Background

Madagascar’s remarkable biodiversity is threatened, and accessible data regarding the distribution, habits, and conservation status of endemic species are key to effective conservation planning^1^. Isotopic research with modern biological materials from the island has helped both to characterize endemic biodiversity and to identify ecological interactions that affect the spatial distribution and abundance of species. For example, researchers have used isotopic data to confirm photosynthetic pathways of endemic plants^2^, identify plant and animal responses to human activities such as forest fragmentation^3–5^, investigate diets of both endangered and introduced animals^6–8^, evaluate spatial partitioning and movement of animals among habitats^7,9,10^, and infer the structure of food webs within terrestrial, freshwater, and nearshore marine environments^11–15^. Isotopic data from modern material can also be integrated with data for ancient organisms to investigate resources used by extinct taxa^16–19^ and reconstruct past changes in the behavior of extant animals^4,20^.

To date, publication of isotopic data has been somewhat haphazard. Some researchers have provided minor compilations of relevant raw isotope data in supplementary files (e.g.^19,21,22^). However, the metadata structure of these partial compilations tends to vary according to specific research questions and among research groups, which hinders reuse. We present the open-access IsoMad (Isotopic Data of Madagascar) database, which is the first compilation to include the majority of known isotopic data for modern materials on the island and in the coastal marine environment. This database follows from several motivations that include the FAIR data principles^23^. First, the initiative eases data accessibility by compiling data that were previously difficult for some researchers to access given journal paywalls, or because authors presented data only as text and summary statistics. We also have >5,000 previously unpublished measurements. Second, the structure of IsoMad makes the data easily searchable and accessible to users who are relatively unfamiliar with the literature. Consequently, the compilation should facilitate future isotopic research by making it easy to identify both research gaps and opportunities for using various isotope systems to answer a given research question. This initial unveiling of IsoMad is meant to be the first step in an ongoing initiative, where researchers will be able to continue adding to the database in perpetuity. It is our hope that the structure of the compilation will serve as a guide for more complete data reporting in future publications.

## Methods

Dataset S1 includes data from multiple different isotopes (δ^2^H, δ^13^C, δ^15^N, δ^18^O, δ^34^S, and ^87^Sr/^86^Sr), from various plant parts (e.g., leaves and fruit), animal tissues (e.g., fur, muscle, bone, and feathers), other organic material (e.g., feces), and water samples that were collected within the past 150 years. Detailed metadata are provided in Dataset S2. We did not include measurements from living archives (e.g., δ^13^C and δ^18^O data from tree ring records spanning hundreds of years) given that these are better suited for a separate database for older specimens that we are in the process of compiling.

The assemblage of published isotopic data from modern materials took place between November 2021 and November 2023. We relied on current professional networks, bibliographies, and internet search engines (e.g., Google Scholar) to locate relevant publications. Search terms included different combinations of keywords such as “Madagascar,” “stable isotope,” “isotopic,” “nitrogen,” “carbon,” and “ecology.” For all journal articles and book chapters published within the past 20 years, we contacted the corresponding authors to request published data and gather outstanding metadata. Not all authors responded or were willing or able to share their published data; a list of the publications that describe data or include summary data and are currently not included in IsoMad is provided in Dataset S3. In addition to published data, we were able to include a relatively large number of previously unpublished isotopic data. Please see Supplementary Information - Methods S1 for details regarding the pre-treatment and analysis of the samples that resulted in these data.

Each entry in Dataset S1 was assigned a site name and georeferenced using decimal degrees in the WGS84 datum system. For freshwater and terrestrial entries, elevation (m above sea level), mean annual precipitation (MAP, mm/yr), and distance to closest coast (km) were estimated using QGIS 3.10.2. Elevation was sampled from the GEBCO-terrestrial raster, and MAP was extracted from the WorldClim2.1 30 s bio12 raster^24^. Distance to the closest coast was calculated by applying the NNJoin tool to sample locations and a trimmed outline of the island reprojected on the EPSG:8441 – Tananarive / Laborde Grid.

Each row is an entry for a unique specimen. Collection date and taxonomic description of each entry are specified as precisely as possible; uncertainties in collection date are indicated by ranges of years. The taxon sampled for analysis is typically identified at least to Kingdom and down to species whenever possible. However, some entries lack taxonomic description beyond common name or local Malagasy name (given in italics) or are mixtures of multiple materials (e.g. particulate organic matter) that cannot be assigned a taxon. Additional species attributes include specification of environment (e.g., “terrestrial” vs. “marine”), plant photosynthetic pathway (“C3”, “C4” or “CAM”), and species status for terrestrial taxa (endemic or introduced, as indicated on the “Global Register of Introduced and Invasive Species – Madagascar”^25^).

Individual specimen attributes are also included to the highest degree possible based on publications and the unpublished notes of authors. This includes sex, body mass (kg), age category, collection method, collection setting, material type (e.g., “feather” vs. “leaf”), element (e.g., specifying bone or muscle component), subsample (for incremental or serially sampled specimens), and additional attribute notes. Given that some entries include isotopic data from multiple materials belonging to a single individual (e.g., fur, bone, and muscle from the same animal), isotopic data in the main material type groups are separated into multiple fields (e.g., “fur δ^13^C” and “muscle δ^13^C”). All stable isotope data are presented relative to international standards (δ^2^H_VSMOW_, δ^13^C_VPDB_, δ^15^N_AIR_, δ^18^O_VPDB_ or δ^18^O_VSMOW_, δ^34^S_VCDT_), and elemental weight %C:N values have been converted to atomic C:N values for consistency. We report water δ^18^O values relative to VSMOW and δ^18^O values from all other materials relative to VPDB. Full references are provided for each isotope system (e.g., “d15N_Source_Reference” versus “d34S_Source_Reference”) for each data entry. This helps clarify sources for specimens that have had isotope data for different elements (e.g., C and N) published in different publications. Additional fields for combinations of material types and isotope systems (e.g. plant δ^18^O values) not yet represented will be added to the compilation and associated metadata descriptions as needed during future updates.

## Data Records

**Fig. 1:**
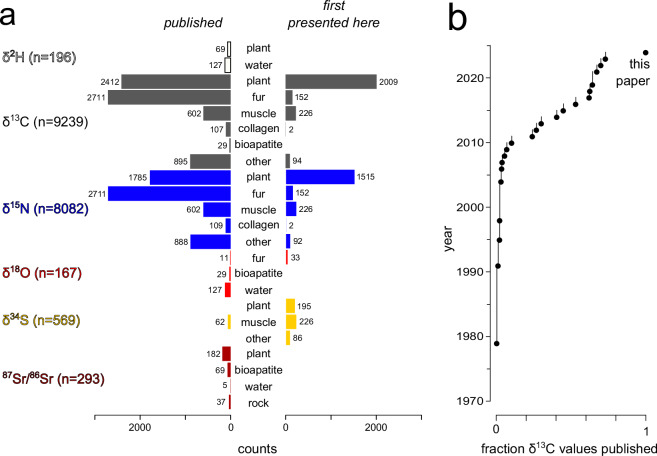
Numbers of isotopic measurements separated according to isotope system, material type and whether or not data were previously published (**a**), and the publication history of the 9,239 δ^13^C values (**b**), which mirrors that of δ^15^N values (δ^13^C and δ^15^N data are typically acquired at the same time). The accumulation of δ^13^C values over the past 50 years in frame b is presented as an empirical cumulative distribution function, which jumps up by x/N at each year when data were published, where N is the total number of published data entries and x is the total number published in the given year.

The IsoMad compilation currently includes 18,578 isotopic measurements from 9,508 specimens; 5,010 of the measurements for 2,725 specimens are reported here for the first time (Fig. 1a)^26^. Most data are δ^13^C and δ^15^N values (Fig. 1a), with just 1,062 measurements (5.7% of total measurement number) from other isotope systems (H, S, O, & Sr). Most data in the compilation were published within the past 15 years, but the collection history of δ^13^C data (paralleling that of δ^15^N data during recent decades) extends over four decades (Fig. 1b). It is only since 2017 that researchers have published δ^34^S and ^87^Sr/^86^Sr data from biological materials collected on the island.

**Fig. 2:**
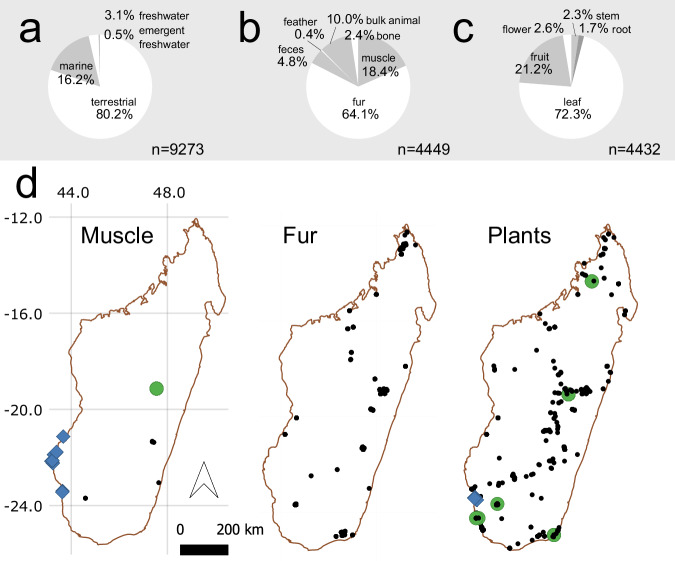
Pie charts showing the relative breakdown of all database entries categorized according to environment (**a**), animal tissue types (**b**), and plant tissue types (**c**), and maps showing the spatial distributions of sampling locations for select material types (**d**), where blue diamonds = marine, green circles = freshwater, black circles = terrestrial. We note that the number of data for plant tissue samples (n = 4,432 entries) and animal tissue samples (n = 4,449) do not sum to the database total (n = 9,508); this is because the database also includes other organisms, such as fungi, and specimens not assigned to Kingdom. Also note that frame b excludes 12 individuals that have data for multiple tissue types, that frame c does not include plant tissues that were very rare (e.g., bark, husk, pod, seed), and that only sites with coordinates known within 50 km are shown in frame d.

Most specimens in the compilation come from terrestrial organisms (Fig. 2a). Fur and muscle are the most commonly analyzed animal tissues (Fig. 2b), and leaves are the most commonly analyzed plant tissues (Fig. 2c). These specimens have a wide geographic distribution (Fig. 2d), although data are relatively limited from much of the western and northeastern parts of the island.

IsoMad is a partner of the IsoMemo network (isomemo.com). The compiled database (Datasets S1-3) is available for download through the IsoMad data community on the Pandora data platform^26^. Data are also accessible via online software developed within the Pandora and IsoMemo initiatives, which facilitates data queries, visualization, and analysis. The IsoMad data community is intended as a general warehouse of isotopic data from Madagascar and in the future may include additional datasets from ancient specimens and continuous archives (e.g., sequentially sampled speleothems). Updates to IsoMad will be made at least annually by both existing administrators (S.H., B.C., & R.F.) and future administrators who can contribute updates directly through the Pandora data platform. Researchers are encouraged to send new datasets to the existing administrators and inquire if interested in becoming administrators.

## Technical Validation

Following data compilation, all sample attribute data were checked and modified as needed for consistency. This involved correcting misspelled or outdated taxonomic names, standardizing the spelling of site names, and confirming geographic coordinates. Given that the accuracy of coordinates varies according to collection method, we included a field (“Coordinate_Type”) to specify the method associated with each entry (e.g., “collected with GPS”, “reported by author”, or “estimated from map”). We also included a field to indicate imprecise site coordinates (“Site_Radius_ > 50_km”).

Previously unpublished data presented in this compilation for the first time are assigned a unique “Analysis_Code”; detailed sample collection and analytical methods for these entries are provided in Supplementary Information – Methods S1. Sample pretreatment and analysis can both impact isotopic data, but these impacts should be relatively minor (<1‰) in most cases^27–30^.

## Usage Notes

The IsoMad compilation of isotopic data from modern materials can be used to identify drivers of isotopic variability among primary producers, evaluate foraging niches of endemic and introduced animals, and serve as a modern reference when studying the subfossil record. As part of the Pandora & IsoMemo initiatives, data from IsoMad are connected with an R-based toolkit of applications for various types of analysis, including spatio-temporal modeling (https://github.com/Pandora-IsoMemo). We briefly illustrate potential uses of the data compilation through two examples involving data from entries 1 through 8,327: (1) Estimations of terrestrial consumer diets based on δ^13^C data (using AverageR & ReSources); and (2) An exploration of the influences of abiotic and biotic variables on plant and mouse lemur (*Microcebus* spp.) δ^15^N values (using the AverageR, OperatoR, and Bayesian Model Selection under Constraints (BMSC) apps).

### Example 1

**Fig. 3:**
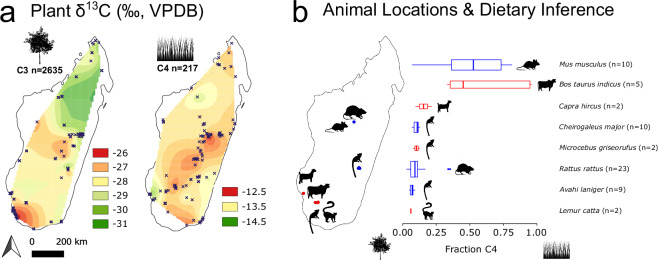
Maps of Madagascar showing interpolated C_3_ and C_4_ plant δ^13^C values based on data from the marked collection sites (**a**), locations of selected mammals with bone collagen δ^13^C data, and the inferred contribution of carbon from C_4_ plant protein in the diet of each group (**b**). Frame a includes δ^13^C values only from plants with known collection times (including specimens collected since 1880) and sites identified to within 50 km. Dietary contributions in frame b were estimated using ReSources, as described in the main text. Box plots have widths scaled to sample size; boxes illustrate interquartile ranges and whiskers extend to minimum/maximum points that fall with 1.5 times the interquartile ranges. Boxes are colored according to region, with blue = Central Highlands and red = southwest Madagascar.

Inferring consumer diet using isotopic data requires (1) knowing different possible dietary sources, and (2) confirming these possible sources are isotopically distinct. Although many types of organisms (e.g., anthropods and fungi) are underrepresented in the IsoMad database, the compilation improves our ability to evaluate diet, such as in the nearshore marine environment around Toliara in southwestern Madagascar (Fig. S1). Given the extensive spatial coverage of isotopic data from terrestrial plants, we are also able to estimate the degree to which consumers from different parts of the island rely on C_3_ plants (mostly woody taxa) and C_4_ plants (mostly grasses)^31^. We assumed for simplicity that the contribution of dietary carbon from CAM succulent plants was minimal. It is possible to use AverageR to generate interpolated surfaces of plant δ^13^C values that can then be used to extract estimated average δ^13^C values for both C_3_ and C_4_ plants at particular sites across the island (Fig. 3a). We corrected plant δ^13^C values for isotopic changes in atmospheric CO_2_ since the Industrial Revolution (the Suess Effect^32^) according to collection year^16^. We emphasize that it is essential that this type of correction is considered during future comparisons, especially those involving δ^13^C values from both modern and ancient material. As expected, there is considerable geographic variability in C_3_ plant δ^13^C values, with lower values in mesic central and northern Madagascar, and higher values in the arid southwest. This reflects differences in water availability as well as physiological differences among plants growing in the different regions^33^. In contrast, there is comparatively little geographic variability in C_4_ plant δ^13^C values, which is consistent with studies elsewhere^34–36^.

We used the Bayesian mixing model called ReSources^37^, an updated version of a previously published mixing model called FRUITS^38^, to infer the fraction of carbon from C_4_ plants in the diet of introduced murid rodents and bovids, as well as endemic lemurs collected from sites in the Central Highlands and relatively arid Southwest. We worked exclusively with bone collagen and used the locations of where bones were collected (Fig. 3b) to extract local estimates of C_3_ and C_4_ plant δ^13^C values (Fig. 3a). Estimated contribution of C_3_ and C_4_ plants to the diet of each species, summarized in Fig. 3b, were derived by independently modeling the proportion of carbon from C_4_ plants in the diet of each individual. We assumed that collagen carbon is derived only from dietary protein C and accounted for the offset in δ^13^C values between consumer collagen and dietary protein through an estimated constant correction of 5.8 ± 1.0‰ (based on a controlled feeding experiment with *Rattus* sp.^39^). Each consumer δ^13^C value was also assumed to have an associated uncertainty of 0.5‰. Consistent with previous work on Madagascar^4,7,20,40^, modeled data suggest that all four lemur species primarily consume C_3_ foods while introduced mice (*Mus musculus*) and cows (*Bos taurus indicus*) consume a fair amount of C_4_ plants. Introduced goats (*Capra hircus*) and murid *Rattus* tend to get more of their food from C_3_ plants, which likely indicates foraging on forest-derived foods rather than grassy biomes. This supports the concern that introduced animals may negatively impact endemic forest-dwelling animals through competition and possibly predation^7,41^.

### Example 2

**Fig. 4:**
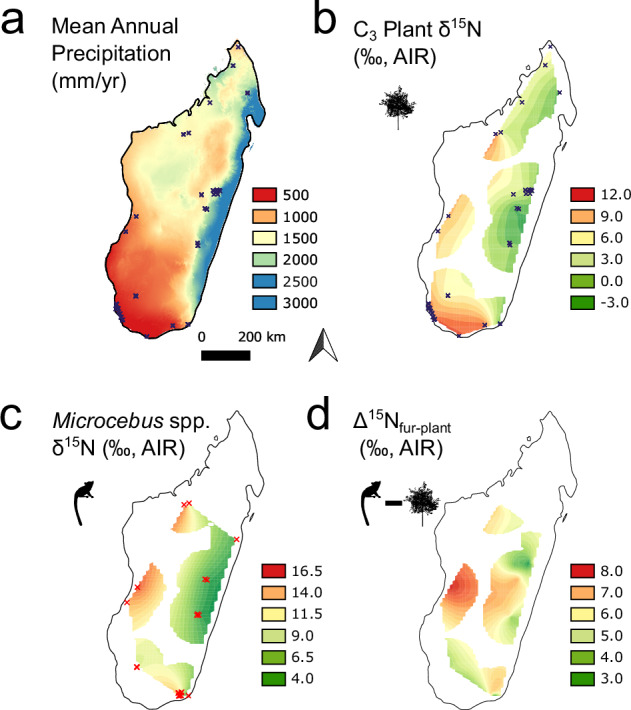
Interpolated surfaces of mean annual precipitation (MAP) (**a**), δ^15^N values for terrestrial C_3_ plant tissues not belonging to members of Fabaceae (n = 2,441) (**b**), δ^15^N values for *Microcebus* spp. fur (n = 1,246) (**c**), and differences between *Microcebus* spp. fur and C_3_ plant tissue δ^15^N values (Δ^15^N_fur-plant_) from the same regions (**d**). MAP data are taken from WorldClim 2.1^24^. Collection site was known to within 50 km for plant and lemur data entries. The δ^15^N surfaces are masked to within 150 km of each collection site (marked with an “x” in frames a-c) and were generated using a Bayesian approach with constant interpolation in AverageR and OperatoR.

Additional inference regarding consumer diet is possible based on the nitrogen isotope content of consumer tissue. Geographic variation in plant δ^15^N values complicates the interpretation of consumer δ^15^N values from different ecoregions^42,43^ but also gives opportunities to learn about the microhabitat use of particular taxa^4,17^. Based on reviews of plant δ^15^N data^44–47^ and previous work on Madagascar^40^, we expect that soil moisture availability, and related variables like mean annual precipitation (MAP), explain the majority of the spatial variation in plant δ^15^N values across the island. Indeed, as generally expected, application of the Bayesian spatial smoothed model AverageR^48^ to the IsoMad compilation (including samples from a variety of years and seasons) illustrates how increasing MAP is associated with lower C_3_ plant δ^15^N values (Fig. 4a,b).

Based on a past study of spatial variation in mouse lemur (*Microcebus* spp.) fur δ^15^N values^40^, we expect isotopic variability in the fur of these C_3_ plant consumers (Fig. 3b) to closely match that of C_3_ plant δ^15^N values. Indeed, geographic differences in δ^15^N values are similar for plants and fur (Fig. 4b-c). However, apparent differences between fur and plant δ^15^N values (Δ^15^N_fur-plant_) are quite variable and range from ~3 to 8‰ across the interpolated surfaces of δ^15^N values (Fig. 4d). A relatively consistent and positive Δ^15^N_fur-plant_ is expected^49^ given that mouse lemurs consume a mix of plant and animal matter, primarily fruit and arthropods^50^. Some species or populations of mouse lemurs might eat relatively more animal matter than others, and this could impact estimated Δ^15^N_fur-plant_ values by up to ~3‰^51,52^. Variation in Δ^15^N_fur-plant_ values can also be explained by different collection times for fur and plant samples, as well as interpolation of plant values across diverse environments. Co-occurring plants can have variable δ^15^N values due to a variety of factors^10,40^, and there can be considerable variability in plant δ^15^N values among adjacent microhabitats^10,40,53^. We used BMSC, a Bayesian regression model selection algorithm^54^, to identify the relative influence of MAP, coastal proximity, and plant part on plant δ^15^N values (Supplementary Information – Usage Notes S1, Fig. S2). This example highlights areas for finer-scale investigation as well as some of the complexity associated with interpreting consumer δ^15^N values.

### Supplementary information

Supplementary Information

## Data Availability

The statistical analysis and modeling employed for examples given in the Usage Notes was done using R packages developed within the Pandora & IsoMemo initiatives^38,48,54^. The source code for all of these is available for download on GitHub: AverageR (https://github.com/Pandora-IsoMemo/DSSM), BMSC (https://github.com/Pandora-IsoMemo/bmsc), and ReSources (https://github.com/Pandora-IsoMemo/resources). These can be run online or locally (https://github.com/Pandora-IsoMemo/drat) as Shiny apps^55^. For modeling reproducibility, a full description of model options is available through the IsoMad data community (https://pandoradata.earth/organization/isomad-isotopic-data-of-madagascar), which is stored on the Pandora data platform. This platform is based on the CKAN open source data management system (https://ckan.org/) and is hosted by the Max Planck Computing and Data Facility.
