# Supplementary material for: Introducing IsoMad, a compilation of isotopic datasets for Madagascar
Source: Sci Data. 2024 Aug 9;11:857. doi: 10.1038/s41597-024-03705-2 (PMC11316086; doi:10.1038/s41597-024-03705-2)
Supplement: Supplementary file 1 — Supplementary Information [file 41597_2024_3705_MOESM1_ESM.pdf]

Supplementary Information for “Introducing IsoMad, a compilation of isotopic datasets for Madagascar”

Table of Contents

Pages 2-6.....................Methods S1
Pages 7-8.....................Usage Notes S1
Pages 9-10...................Supplementary Information References
Pages 11-13.................Data Sources

Methods S1.

The following includes details regarding the pretreatment and analysis of samples that yielded new data in the compilation.

Pretreatment Code: 1
Sites: Ranomafana, Berenty, Tsinjoarivo, Masoala
Contributors: Brooke Crowley, Andrea Baden, Alison Jolly, Mitchell Irwin, Marina Blanco, Timothy Eppley

Sample pre-treatment followed Crowley et al. [1-3]. Any notable debris was manually removed from fur samples using metal forceps. Samples were then placed on individual petri dishes and washed with methanol. Liquid residue was drained, and samples were allowed to air-dry overnight.

For most samples, multiple entire fur strands were shoved into tin boats to obtain appropriate sample mass. However, dwarf lemur fur samples were sectioned into 0.5-cm increments. Samples were either analyzed at (1) the University of California Santa Cruz (UCSC) Stable Isotope Laboratory in Santa Cruz, California USA or (2) the Stable Isotope Biogeochemistry Facility at the University of Cincinnati in Cincinnati Ohio (UC).

At UCSC, approximately 0.7 mg of fur was analyzed on a Finnigan Thermo Electron Delta+XP continuous flow system (Bremen, Germany) connected to a Carlo Erba elemental analyzer (Milan, Italy) via a Conflo III interface (Valencia California, USA. Two reference materials were analyzed along with the samples: homogenized gelatin (PUGEL) and International Atomic Energy Agency (IAEA) acetanilide. Various masses of PUGEL were used to account for sample size (linearity) at the beginning of the run, and additional PUGEL replicates were used to correct for drift throughout each run. Analytical precision was monitored using acetanilide. Based on other work in the lab [1-3], precision (±1 SD) is estimated to have been <0.2‰ for $\delta^{13}C$ was and <0.1‰ for $\delta^{15}N$.

At UC, approximately 0.45 mg of fur was analyzed on a Costech Elemental Analyzer connected to a Thermo Scientific Delta V IRMS (Bremen, Germany) via a Costech Conflo IV interface (Valencia California, USA). Four reference materials were analyzed along with the samples: Powdered caffeine; US Geological Survey (USGS) 41; PUGEL; and powdered glycine. Data were corrected for linearity and drift using powdered caffeine (various masses analyzed at the beginning of the run and then additional replicates with masses more similar to samples were interspersed throughout the run). We accounted for scale using caffeine and USGS41 (n=4 total) analyzed at the beginning and end of the run. Analytical accuracy was evaluated using PUGEL (n=3) and glycine (n=3) that were interspersed across each run. Precision was monitored using all four reference materials. Based on other work in the lab [1-3], accuracy and precision are estimated to have been ca. 0.3‰ for carbon and <0.1‰ for nitrogen.

Pretreatment Code: 2
Sites: Mandena, Ranomafana, Tsimanampetsotsa, Ambohimahavelona, Andrevo, Ranobe
Contributors: Brooke Crowley, Timothy Eppley, Andrea Baden, Steven Goodman, Sean Hixon

Dried plant samples were processed following Crowley et al. [1-3]. Several leaves (or flowers, fruits, or seeds) were homogenized into a powder using an agate mortar and pestle. This process was assisted by freezing samples in liquid nitrogen.

Samples were analyzed at the Stable Isotope Biogeochemistry Facility at the University of Cincinnati in Cincinnati Ohio (UC). We analyzed C and N isotopes separately. First, for carbon, approximately 2 mg of each powdered sample were weighed into a tin boat and combusted on a Costech Elemental Analyzer connected to a Thermo Scientific Delta V IRMS (Bremen, Germany) via a Costech Conflo IV interface (Valencia California, USA). We then weighed out sample-specific masses for nitrogen analyses (informed by the weight %N in each sample). Data were corrected for linearity and drift using powdered caffeine (various masses analyzed at the beginning of the run and then additional replicates with masses more similar to samples were interspersed throughout the run). We accounted for scale using caffeine and either corn starch (for carbon) or US Geological Survey (USGS) 41 (for nitrogen). Two replicates of these references were included at the beginning and end of each run (n=4 total). Analytical accuracy was evaluated using soy flour (n=3) and powdered glycine (n=3) that that were interspersed across each run. Precision was monitored using all four reference materials. Based on historic performance of the lab, accuracy and precision across runs were <0.1‰ for both isotopes.

Pretreatment Code: 3
Sites: Cap Sainte Marie, Mandena
Contributors: Brooke Crowley, Elizabeth Kelley, Timothy Eppley

Dried bone samples were processed following Crowley et al. [4]. Samples were soaked in 0.5N HCl at 4°C until gummy. They were rinsed 5x with ultrapure water and then repeatedly sonicated in petroleum ether until all visible lipids were removed. They were again rinsed 5x with ultrapure water and freeze dried. Samples were analyzed at the Stable Isotope Biogeochemistry Facility at the University of Cincinnati in Cincinnati Ohio (UC). Approximately 0.4 mg of each pre-treated and dried bone were weighed into a tin boat and samples were then combusted on a Costech Elemental Analyzer connected to a Thermo Scientific Delta V IRMS (Bremen, Germany) via a Costech Conflo IV interface (Valencia California, USA). Four reference materials were analyzed along with the samples: Caffeine; US Geological Survey (USGS) 41; PUGEL; and glycine. Data were corrected for linearity and drift using powdered caffeine (various masses analyzed at the beginning of the run and then additional replicates with masses more similar to samples were interspersed throughout the run). We accounted for scale using caffeine and USGS41 (n=4 total) analyzed at the beginning and end of the run. Analytical accuracy was evaluated using PUGEL (n=3) and glycine (n=3) that were interspersed across the run. Precision was monitored using all four reference materials. Based on historic performance of the lab, accuracy and precision across runs were <0.1‰ for both isotopes.

Pretreatment Code: 4
Site: Beza Mahafaly
Contributors: James Loudon, Matt Sponheimer

Plant samples were collected at the Beza Mahafaly Special Reserve (23º30'S latitude, 44 º 40'E longitude), located in southwest Madagascar. Plant samples were placed in labeled bags that included, location of collection, plant organ, plant species name, and local Malagasy plant name. Each sample was wiped clean of any dust or particulate. Plant samples were desiccated fully at the field site in a camp oven. All samples were transported to the University of Colorado, Boulder and further dried in a benchtop oven at 40°C for 24 hours. All plant samples were ground into a fine powder using a mortar and pestle. Approximately 2.5 mg of plant sample powder was placed in tin capsules.

Tin capsules were combusted in an elemental analyzer (Carlo-Erba, Milan, Italy) and analyzed for stable carbon and nitrogen isotope abundances using a flow-through inlet system on a continuous flow isotope ratio mass spectrometer (Finnigan, Bremen, Germany). $^{13}C/^{12}C$ and $^{15}N/^{14}N$ ratios are expressed in delta (δ) notation in parts per thousand or per mil (‰) relative to the Vienna Peedee Belemnite and atmospheric N_2 standards. All samples for this study were analyzed during one analytical run for which standard deviations of replicate measurements of internal standards (protein and yeast) were <0.1‰ for both $\delta^{13}C$ and $\delta^{15}N$ values.

Pretreatment Code: 5
Site: Toliara
Contributor: Loïc Michel

As soon as possible after collection, animals were dissected to separate soft and non-metabolically active tissues (e.g. muscle, tegument) or, when body size was small, were used whole [5]. Obtained samples were oven-dried at 50°C for 72 hours. They were subsequently ground to a homogeneous powder using mortar and pestle or a mixer mill (MM301, Retsch, Haan, Germany) depending on toughness. Samples containing hard inorganic carbon parts that could not be physically removed were acidified by exposing them to HCl vapors for 48h in an airtight container [6]. Stable isotope ratio measurements were performed via continuous flow - elemental analysis - isotope ratio mass spectrometry (CF-EA-IRMS) at University of Liège (Belgium), using a vario MICRO cube C-N-S elemental analyzer (Elementar Analysensysteme GMBH, Hanau, Germany) coupled to either an IsoPrime100 or a precisION isotope ratio mass spectrometer (Isoprime, Cheadle, United Kingdom). Isotopic ratios were expressed using the widespread δ notation (Coplen 2011), in ‰ and relative to the international references Vienna Pee Dee Belemnite (for carbon), atmospheric air (for nitrogen) and Vienna Canyon Diablo Troilite (for sulfur). IAEA (International Atomic Energy Agency, Vienna, Austria) certified reference materials sucrose (IAEA-C-6; $\delta^{13}C = -10.8 \pm 0.5$‰; mean ± SD), ammonium sulfate (IAEA-N-1; $\delta^{15}N = 0.4 \pm 0.1$‰; mean ± SD ; or IAEA-N-2; $\delta^{15}N = 20.4 \pm 0.1$‰; mean ± SD) and silver sulfide (IAEA-S-1; $\delta^{34}S = -0.3$‰ ; or IAEA-S-1; $\delta^{34}S = 22.6$‰) were used as primary analytical standards. Sulfanilic acid (Sigma-Aldrich; $\delta^{13}C = -25.6 \pm 0.4$‰; $\delta^{15}N = -0.13 \pm 0.4$‰; $\delta^{34}S = 5.9 \pm 0.5$‰; means ± SD) was used as secondary analytical standard. Standard deviations on multi-batch replicate measurements of secondary and internal lab standards (amphipod crustacean muscle, seabass muscle or seagrass leaves, according to sample nature) analyzed interspersed with samples (one replicate of each standard every 15 analyses) were typically 0.2‰ for both $\delta^{13}C$ and $\delta^{15}N$ and 0.4‰ for $\delta^{34}S$.

Pretreatment Code: 6
Site: Tsimanampetsotsa, Marojejy, Mandena, Berenty, Masoala
Contributors: McAntonin Andriamahaihavana, Iris Dröscher, Timothy Eppley, Jörg Ganzhorn, Dean Gibson, Marni LaFleur, Erik Patel, Delaïd C. Rasamisoa, Yedidya Ratovonamana, Christoph Reisdorff, Natalie Vasey

Two grams of dried plant biomass were ground in a Retsch MM200 mill (Retsch GmbH, Hann, Germany) and subsequently stored dry in 2 mL Eppendorf tubes until further analysis. Samples of around 2 mg were weighed into tin cups (HEKAtech, Germany) for analysis in a stable isotope ratio mass spectrometer (nu Horizon, Nu Instruments Limited, UK) linked to an elemental analyzer (EURO-EA 3000, Euro Vector, Italy) in continuous flow configuration (set-up by HEKAtech, Germany).

Data calibration was conducted using the certified standard IAEA-NO-3 Potassium Nitrate ($\delta^{15}N$ = +4.7‰ air N_2, SD 0.2‰) and IAEA-600 Caffeine ($\delta^{13}C$ = −27.8‰ VPDB, SD 0.0‰) [7]. Potassium Nitrate and Caffeine standards were injected every 40 runs. For carbon, samples were measured against CO_2 reference gas that had been calibrated to L-glutamic acid reference materials USGS-40 ($\delta^{13}C$ = −26.4‰ VPDB) and USGS-41 ($\delta^{13}C$ = −37.6‰ VPDB) (International Atomic Energy Agency, Vienna, Austria). We used NIST-certified 2,5-bis(5-tert-butyl-2-benzoxazol-2- yl)thiophene (BBOT; 72.5% C; HEKAtech, Germany) as internal reference material for carbon isotope analysis [8]. BBOT standards were injected every 13 runs. For BBOT, the mean and standard deviation of 40 measurements of $\delta^{13}C$ was -26.1 ± 0.1‰. As a further assessment of precision, we used 259 samples that have been analyzed in duplicate. For these samples, we calculated the difference between the mean per item and the actual measurement. Differences were 0.0 ± 0.1‰ (mean ± SD) for $\delta^{15}N$, and 0.0 ± 0.1‰ for $\delta^{13}C$.

Pretreatment Code: 7
Site: Berenty, Kirindy
Contributors: Andrius Garbaras

Dried plant samples were homogenized into a powder after freezing in liquid nitrogen using an agate mortar and pestle. Stable isotope ratio measurements were performed via continuous flow - elemental analysis - isotope ratio mass spectrometry (CF-EA-IRMS) at Center for Physical Sciences and Technology, (Vilnius, Lithuania), using a Flash EA1112 C-N-S elemental analyzer (ThermoScientific, Bremen, Germany) coupled to Thermo Delta V isotope ratio mass spectrometer (ThermoScientific, Bremen, Germany). Isotopic ratios were expressed using the widespread δ notation [9], in ‰ and relative to the international references Vienna Pee Dee Belemnite (for carbon), atmospheric air (for nitrogen). IAEA (International Atomic Energy Agency, Vienna, Austria) certified reference materials caffeine (IAEA-600; $\delta^{13}C$ = -27.77 ± 0.04‰; mean ± SD), Graphite (USGS24; $\delta^{13}C$ = -16,05 ± 0.04‰; mean ± SD) ammonium sulfate (IAEA-N-1; $\delta^{15}N$ = 0.4 ± 0.1‰; mean ± SD ; or IAEA-N-2; $\delta^{15}N$ = 20.4 ± 0.1‰; mean ± SD) were used as primary analytical standards. Caffeine (Sigma-Aldrich; $\delta^{13}C$ = -32.68 ± 0.15‰; $\delta^{15}N$ = -2.9 ± 0.2‰; means ± SD) was used as secondary analytical standard. Secondary analytical standard was used after every 12 sample measurements.

Pretreatment Code: 8
Site: Toliara
Contributor: Lucas Terrana, Gilles Lepoint, Richard Rasolofonirina, Igor Eeckhaut

Analytical procedure: Fresh mangrove crabs were measured on their cephalothorax, then sexed, and dissected to sample the left cheliped (i.e. first thoracic limb of crabs bearing a pincer or claw or nipper). Internal muscle of the cheliped were extracted, taking attention not to sample cuticle fragments which are carbonated. Samples were dried at 60°C during 48h and then ground using pestle and mortar. Mangrove leaves were dried at 60°C during 48h and then ground using a Retsch MM 301 micro-ball mill (2 min, 25 Hz) to obtain a homogeneous powder, which was weighed and placed into tin capsules. Stable isotope ratio measurements were performed via continuous flow - elemental analysis - isotope ratio mass spectrometry (CF-EA-IRMS) at University of Liège (Belgium), using a vario MICRO cube C-N-S elemental analyzer (Elementar Analysensysteme GMBH, Hanau, Germany) coupled to either an IsoPrime100 isotope ratio mass spectrometer (Isoprime, Cheadle, United Kingdom).

Traceability: Isotopic ratios are presented as δ values (‰), expressed relative to the vPDB (Vienna Peedee Belemnite) standard and to atmospheric N_2 for carbon and nitrogen, respectively. Reference materials were IAEA-N1 ($\delta^{15}N=+0.4 \pm 0.2$‰) and IAEA CH-6 (sucrose) $\delta^{13}C= -10.4 \pm 0.2$‰). Each reference material was measured in duplicate at the beginning and end of each analytical batch.

Uncertainty: Experimental precision (based on the standard deviation of replicates of an internal standard) was 0.3 and 0.4‰ for carbon and nitrogen, respectively.

Usage Notes S1.

Example 1:

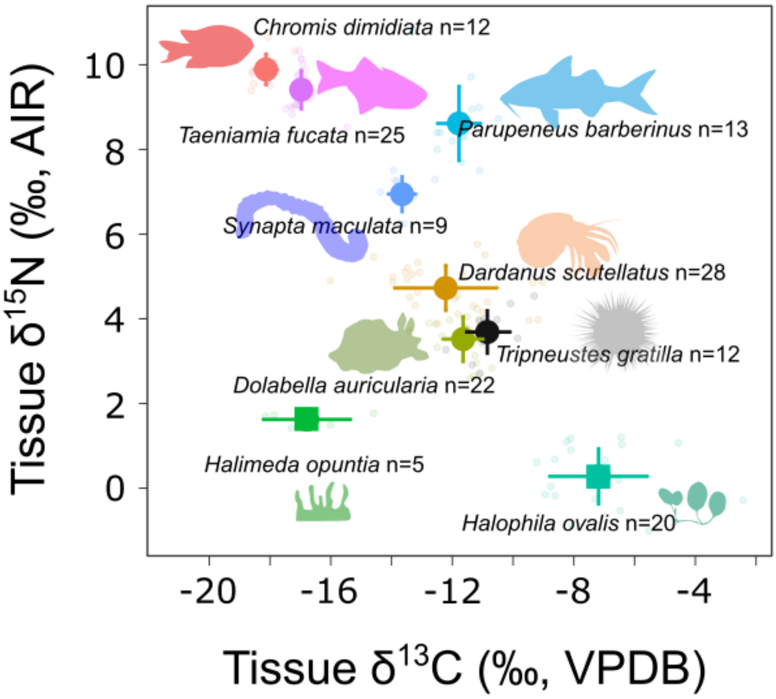

Figure S1. *Illustration of $\delta^{13}C$ and $\delta^{15}N$ values from the tissues of selected primary producers (squares) and consumers (circles) collected from the reef flats surrounding Toliara (n=146). Consumer tissues are muscle, with the exception of tegument analyzed from* Synapta maculata, *and colored large points and lines mark group means and standard deviations. Note that dietary inference through stable isotope analysis of consumer tissues relies necessarily on the distinct isotopic composition of different dietary sources, and IsoMad makes it easy to explore diverse sources. This example highlights the distinct $\delta^{13}C$ values of primary producer tissues and a range of trophic levels reflected in consumer tissue $\delta^{15}N$ values [10-12]. Some of these groups may provide useful endmembers for future work involving dietary inference from stable isotope data.*

Example 2:

Increasing MAP is generally associated with lower C_3 plant $\delta^{15}N$ values (Fig. 4A-B). However, there remains a large fraction of variation in plant $\delta^{15}N$ values that is not explained by MAP. Previous research has identified significant effects on plant $\delta^{15}N$ values from differences in mean annual temperature[13, 14], mycorrhizal associations[14, 15], photosynthetic pathway[16, 17], coastal proximity[18, 19], plant part[20], tissue [N][14, 21], plant community composition[22, 23], human activities (e.g., manuring)[24], soil quality[13, 25], and topography[13].

For example, as expected, the observed negative relationship between water availability and plant $\delta^{15}N$ values is stronger in C_3 plants than in C_4 plants (Fig. S2). Additionally, plants from poorly drained wetlands (e.g., those from Mandena with MAP of ~1200 mm/yr) tend to be relatively depleted in ^{15}N. Also, given that members of Fabaceae are frequently associated with N-fixing bacteria (tending to lower foliar $\delta^{15}N$ values) we expected that removing specimens from this family (n=378, spanning the full aridity gradient) from analysis would strengthen the observed relationship between $\delta^{15}N$ and MAP. However, we found the

adjusted r^2 of 0.68 unchanged, which may follow from the diverse taxa represented in this family and a range of mycorrhizal associations spread across other families.

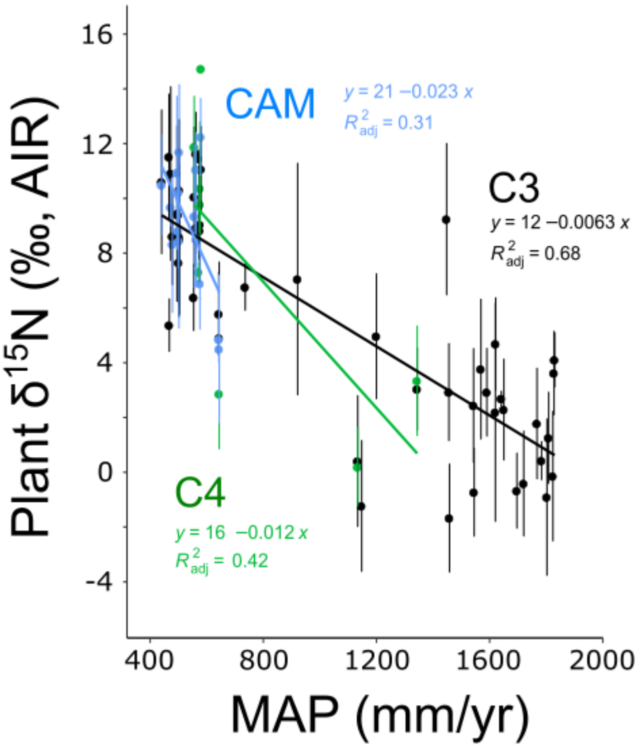

Figure S2. *Observed relationships between MAP and terrestrial plant $\delta^{15}N$ values according to plant photosynthetic pathway. Plant data are separated and color coded according to plant photosynthetic pathway. Points and whiskers mark the mean and standard deviation, respectively, of plant $\delta^{15}N$ values at each site. MAP data are taken from WorldClim 2.1[26].*

We used BMSC, a Bayesian regression model selection algorithm [27], to further explore the effects of two continuous predictors (MAP & Coast_Distance) and two categorical factors (Plant_Pathway & Material) on plant $\delta^{15}N$ values. This modeled dataset includes 2,855 $\delta^{15}N$ measurements from terrestrial plants with known photosynthetic pathway, specified plant part, and collection site known within 50 km. The associated model file can be accessed on Pandora (https://pandoradata.earth/dataset/isomad-modern-biological-material) and viewed with BMSC. The Bayesian Information Criterion suggests that the most parsimonious model includes all four of the aforementioned predictors (BIC=7193.14). As expected, the model suggests that plants both closer to the coast and those from relatively arid locations have tissues relatively enriched in ^{15}N. Model parameters also indicate that roots are typically enriched in ^{15}N relative to stems and flowers and that C_3 plant tissues tend to be enriched in ^{15}N relative to those of C_4 plants.

Supplementary Information References

1. Crowley, B., et al., *Stable isotopes document resource partitioning and effects of forest disturbance on sympatric cheirogaleid lemurs.* Naturwissenschaften, 2013. **100**(10): p. 943-956.
2. Crowley, B.E., E. Rasoazanabary, and L.R. Godfrey, *Stable isotopes complement focal individual observations and confirm dietary variability in reddish–gray mouse lemurs (*Microcebus griseorufus*) from southwestern Madagascar.* American Journal of Physical Anthropology, 2014. **155**(1): p. 77-90.
3. Crowley, B.E., et al., *Explaining geographical variation in the isotope composition of mouse lemurs (*Microcebus*).* Journal of Biogeography, 2011. **38**(11): p. 2106-2121.
4. Crowley, B.E., et al., *Isotopic evidence for niche partitioning and the influence of anthropogenic disturbance on endemic and introduced rodents in central Madagascar.* The Science of Nature, 2018. **105**(7): p. 1-13.
5. Mateo, M.A., et al., *Effects of sample preparation on stable isotope ratios of carbon and nitrogen in marine invertebrates: implications for food web studies using stable isotopes.* Oecologia, 2008. **157**: p. 105-115.
6. Hedges, J.I. and J.H. Stern, *Carbon and nitrogen determinations of carbonate-containing solids 1.* Limnology and Oceanography, 1984. **29**(3): p. 657-663.
7. Mueller, P.M., et al., *With a little help from my friends: physiological integration facilitates invasion of wetland grass* Elymus athericus *into flooded soils.* Oikos, 2021. **130**(3): p. 431-439.
8. Loydi, A., et al., *Opposite effects of litter and hemiparasites on a dominant grass under different water regimes and competition levels.* Plant ecology, 2018. **219**: p. 133-144.
9. Coplen, T.B., *Guidelines and recommended terms for expression of stable-isotope-ratio and gas-ratio measurement results.* Rapid Communications in Mass Spectrometry, 2011. **25**(17): p. 2538-2560.
10. Mittelheiser, L., et al., *Ecomorphology of six goatfish species (Mullidae) from Toliara Reef, Madagascar.* Environmental Biology of Fishes, 2022. **105**(8): p. 1015-1032.
11. Frédérich, B., et al., *Comparative feeding ecology of cardinalfishes (Apogonidae) at Toliara Reef, Madagascar.* Zoological studies, 2017. **56**.
12. Frédérich, B., et al., *Trophic niches of thirteen damselfishes (Pomacentridae) at the Grand Récif of Toliara, Madagascar.* Ichthyological Research, 2009. **56**: p. 10-17.
13. Amundson, R., et al., *Global patterns of the isotopic composition of soil and plant nitrogen.* Global biogeochemical cycles, 2003. **17**(1).
14. Craine, J.M., et al., *Global patterns of foliar nitrogen isotopes and their relationships with climate, mycorrhizal fungi, foliar nutrient concentrations, and nitrogen availability.* New Phytologist, 2009. **183**(4): p. 980-992.
15. Hobbie, E.A. and P. Högberg, *Nitrogen isotopes link mycorrhizal fungi and plants to nitrogen dynamics.* New Phytologist, 2012. **196**(2): p. 367-382.
16. Luo, W., et al., *Higher capability of C3 than C4 plants to use nitrogen inferred from nitrogen stable isotopes along an aridity gradient.* Plant and Soil, 2018: p. 1-11.
17. Swap, R., et al., *Natural abundance of ^{13}C and ^{15}N in C3 and C4 vegetation of southern Africa: patterns and implications.* Global Change Biology, 2004. **10**(3): p. 350-358.
18. Reimchen, T., et al. *Isotopic evidence for enrichment of salmon-derived nutrients in vegetation, soil, and insects in riparian zones in coastal British Columbia*. in *American Fisheries Society Symposium*. 2003. American Fisheries Society.

19. Heaton, T.H., *The 15 N/14 N ratios of plants in South Africa and Namibia: relationship to climate and coastal/saline environments.* Oecologia, 1987. **74**: p. 236-246.
20. Codron, J., et al., *Taxonomic, anatomical, and spatio-temporal variations in the stable carbon and nitrogen isotopic compositions of plants from an African savanna.* Journal of Archaeological Science, 2005. **32**(12): p. 1757-1772.
21. Craine, J.M., et al., *Ecological interpretations of nitrogen isotope ratios of terrestrial plants and soils.* Plant and Soil, 2015. **396**: p. 1-26.
22. de Vries, F.T. and R.D. Bardgett, *Plant community controls on short-term ecosystem nitrogen retention.* New Phytologist, 2016. **210**(3): p. 861-874.
23. Gubsch, M., et al., *Foliar and soil $\delta^{15}N$ values reveal increased nitrogen partitioning among species in diverse grassland communities.* Plant, Cell & Environment, 2011. **34**(6): p. 895-908.
24. Aranibar, J., et al., *Nitrogen isotope composition of soils, C 3 and C 4 plants along land use gradients in southern Africa.* Journal of Arid Environments, 2008. **72**(4): p. 326-337.
25. Kerley, S. and S. Jarvis, *Variation in ^{15}N natural abundance of soil, humic fractions and plant materials in a disturbed and an undisturbed grassland.* Biology and fertility of soils, 1997. **24**: p. 147-152.
26. Fick, S.E. and R.J. Hijmans, *WorldClim 2: new 1-km spatial resolution climate surfaces for global land areas.* International Journal of Climatology, 2017. **37**(12): p. 4302-4315.
27. Fernandes, R., et al., *Deletion/Substitution/Addition (DSA) model selection algorithm applied to the study of archaeological settlement patterning.* Journal of Archaeological Science, 2011. **38**(9): p. 2293-2300.

Data Sources

1. Bamford, A.J., et al., *The status and ecology of the last wild population of Madagascar Pochard* Aythya innotata*.* Bird Conservation International, 2015. **25**(1): p. 97-110.
2. Benstead, J.P. and C.M. Pringle, *Deforestation alters the resource base and biomass of endemic stream insects in eastern Madagascar.* Freshwater Biology, 2004. **49**(4): p. 490-501.
3. Caulier, G., et al., *The diet of the Harlequin crab Lissocarcinus orbicularis, an obligate symbiont of sea cucumbers (holothuroids) belonging to the genera* Thelenota*,* Bohadschia *and* Holothuria*.* Symbiosis, 2014. **62**: p. 91-99.
4. Crowley, B.E. and L.R. Godfrey, *Why all those spines?: Anachronistic defences in the Didiereoideae against now extinct lemurs.* South African Journal of Science, 2013. **109**(1-2): p. 1-7.
5. Crowley, B., et al., *Stable isotopes document resource partitioning and effects of forest disturbance on sympatric cheirogaleid lemurs.* Naturwissenschaften, 2013. **100**(10): p. 943-956.
6. Crowley, B.E., et al., *Stable carbon and nitrogen isotope enrichment in primate tissues.* Oecologia, 2010. **164**(3): p. 611-626.
7. Crowley, B.E., et al., *Isotopic evidence for niche partitioning and the influence of anthropogenic disturbance on endemic and introduced rodents in central Madagascar.* The Science of Nature, 2018. **105**(7): p. 1-13.
8. Crowley, B.E., K.C. McGoogan, and S.M. Lehman, *Edge effects on foliar stable isotope values in a Madagascan tropical dry forest.* PloS one, 2012. **7**(9): p. e44538.
9. Crowley, B.E., E. Rasoazanabary, and L.R. Godfrey, *Stable isotopes complement focal individual observations and confirm dietary variability in reddish–gray mouse lemurs (*Microcebus griseorufus*) from southwestern Madagascar.* American Journal of Physical Anthropology, 2014. **155**(1): p. 77-90.
10. Crowley, B.E., et al., *Explaining geographical variation in the isotope composition of mouse lemurs (*Microcebus*).* Journal of Biogeography, 2011. **38**(11): p. 2106-2121.
11. Crowley, B.E., H.H. Schmidt, and M.S. Vorontsova, *Carbon isotope values for grasses in Madagascar's Central Highlands establish baselines for historical and paleoecological research.* Plants, People, Planet, 2023. **5**(6): p. 869-884.
12. Crowley, B.E., et al., *Strontium isotopes are consistent with low-elevation foraging limits for Henst's goshawk.* Wildlife Society Bulletin, 2017. **41**(4): p. 743-751.
13. Dammhahn, M. and S.M. Goodman, *Trophic niche differentiation and microhabitat utilization revealed by stable isotope analyses in a dry-forest bat assemblage at Ankarana, northern Madagascar.* Journal of Tropical Ecology, 2014. **30**(2): p. 97-109.
14. Dammhahn, M. and P.M. Kappeler, *Scramble or contest competition over food in solitarily foraging mouse lemurs (*Microcebus *spp.): new insights from stable isotopes.* American Journal of Physical Anthropology: The Official Publication of the American Association of Physical Anthropologists, 2010. **141**(2): p. 181-189.
15. Dammhahn, M., C.F. Rakotondramanana, and S.M. Goodman, *Coexistence of morphologically similar bats (Vespertilionidae) on Madagascar: stable isotopes reveal fine-grained niche differentiation among cryptic species.* Journal of Tropical Ecology, 2015. **31**(2): p. 153-164.
16. Dammhahn, M., T.M. Randriamoria, and S.M. Goodman, *Broad and flexible stable isotope niches in invasive non-native* Rattus *spp. in anthropogenic and natural habitats of central eastern Madagascar.* BMC ecology, 2017. **17**(1): p. 1-13.

17. Dammhahn, M., V. Soarimalala, and S.M. Goodman, *Trophic Niche Differentiation and Microhabitat Utilization in a Species-rich Montane Forest Small Mammal Community of Eastern Madagascar.* Biotropica, 2013. **45**(1): p. 111-118.
18. Fourgon, D., G. Lepoint, and I. Eeckhaut, *Assessment of trophic relationships between symbiotic tropical ophiuroids using C and N stable isotope analysis.* Journal of the Marine Biological Association of the United Kingdom, 2006. **86**(6): p. 1443-1447.
19. Frédérich, B., et al., *Trophic niches of thirteen damselfishes (Pomacentridae) at the Grand Récif of Toliara, Madagascar.* Ichthyological Research, 2009. **56**: p. 10-17.
20. Frédérich, B., et al., *Comparative feeding ecology of cardinalfishes (Apogonidae) at Toliara Reef, Madagascar.* Zoological studies, 2017. **56**.
21. Godfrey, L.R., et al., *What did* Hadropithecus *eat, and why should paleoanthropologists care?* American Journal of Primatology, 2016. **78**(10): p. 1098-1112.
22. Heck, L., et al., *Determinants of isotopic variation in two sympatric mouse lemur species from northwestern Madagascar.* The Dwarf and Mouse Lemurs of Madagascar: Biology, Behavior and Conservation Biogeography of the Cheirogaleidae, 2016. **73**: p. 281.
23. Hixon, S., et al., *Drought Coincided with, but Does Not Explain, Late Holocene Megafauna Extinctions in SW Madagascar.* Climate, 2021. **9**(9): p. 138.
24. Hixon, S.W., et al., *Late Holocene spread of pastoralism coincides with endemic megafaunal extinction on Madagascar.* Proc. R. Soc. Lond. B, 2021. **288**(1955): p. 20211204.
25. Hixon, S., et al., *Ecological consequences of a millennium of introduced dogs on Madagascar.* Frontiers in Ecology and Evolution, 2021. **9**: p. 428.
26. Hixon, S.W., et al., *Dogs occupying grassy habitat near protected areas in eastern Madagascar rely on foods from forests.* Plants, People, Planet, 2022.
27. IAEA/WMO, *Global Network of Isotopes in Precipitation*, 2024: The GNIP Database.
28. Kiszka, J.J., et al., *Trophic ecology of common elasmobranchs exploited by artisanal shark fisheries off south-western Madagascar.* Aquatic Biology, 2014. **23**(1): p. 29-38.
29. Kluge, M., et al., *Ecophysiological studies on the vegetation of Madagascar: a δ13C and δD survey for incidence of crassulacean acid metabolism (CAM) among orchids from montane forests and succulents from the xerophytic thorn-Bush.* Isotopes in environmental and health studies, 1995. **31**(2): p. 191-210.
30. Kluge, M., et al., *Crassulacean acid metabolism in* Kalanchoë *species collected in various climatic zones of Madagascar: a survey by $\delta^{13}C$ analysis.* Oecologia, 1991. **88**: p. 407-414.
31. Lepoint, G., et al., *Isotopic ratios and elemental contents as indicators of seagrass C processing and sewage influence in a tropical macrotidal ecosystem (Madagascar, Mozambic channel).* Scientia Marina, 2008. **72**(1).
32. Lepoint, G., et al., *Trophic ecology of the seagrass-inhabiting footballer demoiselle* Chrysiptera annulata *(Peters, 1855); comparison with three other reef-associated damselfishes.* Belgian Journal of Zoology, 2016. **146**(1).
33. Loudon, J.E., et al., *Intraspecific variation in hair $\delta^{13}C$ and $\delta^{15}N$ values of ring-tailed lemurs (*Lemur catta*) with known individual histories, behavior, and feeding ecology.* American Journal of Physical Anthropology: The Official Publication of the American Association of Physical Anthropologists, 2007. **133**(3): p. 978-985.
34. Lührs, M.-L., M. Dammhahn, and P. Kappeler, *Strength in numbers: males in a carnivore grow bigger when they associate and hunt cooperatively.* Behavioral Ecology, 2013. **24**(1): p. 21-28.

35. Mittelheiser, L., et al., *Ecomorphology of six goatfish species (Mullidae) from Toliara Reef, Madagascar.* Environmental Biology of Fishes, 2022. **105**(8): p. 1015-1032.
36. Mortillaro, J.-M., et al., *Trophic functioning of integrated rice-fish farming in Madagascar: Insights from stable isotopes ($\delta^{13}C$ & $\delta^{15}N$).* Aquaculture, 2022. **555**: p. 738240.
37. Rakotondranary, S.J., et al., *Regional, seasonal and interspecific variation in ^{15}N and ^{13}C in sympatric mouse lemurs.* Naturwissenschaften, 2011. **98**: p. 909-917.
38. Reuter, K.E., et al., *Using stable isotopes to infer the impacts of habitat change on the diets and vertical stratification of frugivorous bats in Madagascar.* PloS one, 2016. **11**(4): p. e0153192.
39. Schoeninger, M.J., U.T. Iwaniec, and L.T. Nash, *Ecological attributes recorded in stable isotope ratios of arboreal prosimian hair.* Oecologia, 1998. **113**: p. 222-230.
40. Terrana, L., G. Lepoint, and I. Eeckhaut, *Assessing trophic relationships between shallow-water black corals (Antipatharia) and their symbionts using stable isotopes.* Belgian Journal of Zoology, 2019. **149**.
41. Terrana, L., et al., *The bed and board services of crinoids to their associated fauna: a case study from the Great Reef of Toliara, SW Madagascar.* Community Ecology, submitted.
42. Winter, K., *$\delta^{13}C$ values of some succulent plants from Madagascar.* Oecologia, 1979. **40**(1): p. 103-112.
